# Supplementary material for: Global and Selective Effects of Auditory Attention on Arousal: Insights From Pupil Dilation
Source: J Cogn. 2026 Jan 7;9(1):3. doi: 10.5334/joc.474 (PMC12785711; doi:10.5334/joc.474)
Supplement: Supplementary data. — Statistical analysis of the cue-locked PR (with NoDIS and DIS trials). [file joc-9-1-474-s1.pdf]

Supplementary data

Results section

PR to cues, with distracting sounds

To test the distractor effect on the cue-locked PR in the PAV condition, the mean amplitudes of the PDR were compared in the distractor and no-distractor conditions. No significant distractor effect was found in the PAV condition ($p > .11$ for the six 500-ms time-windows from 0 to 3000 ms) (**Sup Figure 3A**).

To test the distractor effect on the cue-locked PR in the AAV condition, the mean amplitudes of the PR were compared in the distractor and no-distractor conditions. From 500 to 3000ms, the mean amplitude was larger in the distractor than the no-distractor condition (500-1000ms: $Z = 153$, $p < .05$, $r = .40$; 1000-1500ms: $Z = 209$, $p < .0001$, $r = .87$; 1500-2000ms: $Z = 210$, $p < .0001$, $r = .88$; 2000-2500ms: $Z = 207$, $p < .0001$, $r = .85$; 2500-3000ms : $Z = 179$, $p < .01$, $r = .62$) (**Sup Figure 3B**). No significant effect was found before 500 ms (0-500ms: $Z = 104.5$, $p = .51$). These data clearly show a pupil dilation response to distracting sounds in the AAV condition.

Then, to test the task effect on the cue-locked PR to distractor, the mean differences in amplitude between distractor and no-distractor conditions (DIS-NoDIS) were compared in the active and passive conditions. From 500 to 2500ms, the mean DIS-NoDIS difference in amplitude was larger in the active than the passive condition (500-1000ms: $Z = 154$, $p < .05$, $r = .41$; 1000-1500ms: $Z = 175$, $p < .01$, $r = .58$; 1500-2000ms: $Z = 182$, $p < .01$, $r = .64$; 2000-2500ms: $Z = 154$, $p < .05$, $r = .41$) (**Sup Figure 4A**). No significant effect of the task was found on the other time-windows (0-500ms: $Z = 128$, $p = .2$; 2500-3000ms: $Z = 137$, $p = .12$). The pupil dilation response to distractor is larger in the active than in the passive condition.

Finally, to test the cue effect on the cue-locked PR to distractor, the mean differences in amplitude between active and passive conditions (AAV-PAV) of the PR to distractor (DIS-NoDIS) were compared in the informative and uninformative cue conditions. No significant effect of the cue was found on these differences ($p > .14$ for the 6 time-windows) (**Sup Figure 4B**). Bayesian Wilcoxon tests showed no conclusive evidence for or against a cue effect from 1000 to 2000 ms ($0.681 < BF_{10} < 0.746$) and positive evidence for no cue effect from 0 to 1000 ms (0-500ms: $BF_{10} = .248$; 500-1000ms: $BF = .323$) and 2000 to 3000ms (2000-2500ms: $BF = .323$; 2500-3000ms: $BF = .239$). There is no clear evidence for an effect of the cue on the pupil dilation response to task-irrelevant distractors.
